# Fatal police violence near reservations: Interpreting risk in context

**DOI:** 10.1073/pnas.2607272123

**Published:** 2026-04-20

**Authors:** Jerreed D. Ivanich

**Affiliations:** ^a^The Centers for American Indian and Alaska Native Health, University of Colorado Anschutz Medical Campus, Aurora, CO 80045

Police violence is a public health concern in the United States (1). Much of the prior work on police violence has established a firm foundation of racial inequity in police violence, specifically for Black and Latino populations (2). Additionally, most of the work has focused on urban settings (3). These foci have contributed, to some degree, to the lack of attention on police violence among American Indian and Alaska Native (AIAN) populations. The PNAS paper “Heightened risk of fatal police violence in and around reservations for American Indian and Alaska Native Peoples in the United States” by Schwartz et al. (4) begins to fill this important gap and increases visibility of police violence for AIAN peoples living on or near reservations. They highlight that one in 1,800 AIAN men in the United States will die from fatal police violence, which is an elevated risk compared to the national male lifetime risk of one in 2,000 (5). Beyond documenting this important disparity, Schartz et al. highlight the role of place, history, and governance structures in shaping risk.

Studying police violence, specifically, fatal police violence, among AIAN populations has faced an aggregately persistent set of challenges. One major challenge has been the lack of representation of the AIAN population in police mortality and official administrative data (6). Another barrier to this work has been the well-documented misclassification of AIAN peoples in these existing data (7). Finally, most studies have focused on major metropolitan settings. Taken together, these may represent three of the major drivers of the lack of visibility of AIAN populations in available data and thus, the lack of representation in the extant literature of police violence.

Schwartz et al. address these concerns in various meaningful ways. First, these authors use data from the Mapping Police Violence database (8), which is the most robust database of fatal police shootings in the United States across all races/ethnicities and is not state or regionally limited. The primary aim of Schwartz et al. was to assess the role of geography in the risk of police violence (on reservation, within 5 miles of the reservation, within 5 and 10 miles of the reservation, and “far”–greater than 10 miles away from the reservation).

Schwartz et al. also address the broader and vexing issue of racial misclassification. Racial misclassification is a persistent issue in working with national databases for AIANs. This problem extends beyond a measurement issue, as racial and ethnic misclassification has real-world implications for policy and funding appropriations. Estimates suggest that the racial misclassification rates within death certificate records among AIAN peoples hover around 40% (9). Through a rigorous set of sensitivity analyses, Schwartz et al. aimed to address this disturbing problem. They find that between 60% and 78% of AIAN police violence deaths far from the reservations would have had to be misclassified as some other race to explain away the elevated risk of living on or near the reservation. Their sensitivity analysis approach to assessing the scientific soundness of their results can and should be applied to other populations facing high rates of racial misclassification. Their work documents the distribution of fatal police violence among AIAN peoples, and it also raises meaningful questions as to *why risk may be concentrated* on and near reservation settings.

## Geography and Jurisdictional Complexity.

The elevated risks noted on or near reservations constitute a significant geographic finding but should be interpreted as nuanced, complex governance and jurisdictional structures that shape public health safety for AIAN populations (10). For example, law enforcement on and near reservations often involves overlapping jurisdictions, including tribal police departments, county sheriffs, state agencies, and other federal agencies such as the Bureau of Indian Affairs and the Federal Bureau of Investigation. Actors involved are heavily dependent on the historical and contemporary governance policies and geographical considerations. All of which leads to a fragmented and complex set of authorities policing reservation lands.

Complicating this relationship further is the consistent structural disinvestment of AIAN public safety systems. The lack of resources in rural infrastructure has forced many of these law enforcement agencies to become primary responders to public health crises, including, but not limited to, substance use, interpersonal violence, and mental health conditions, rather than other social safety net programs and social workers (11). Thus, increasing law enforcement presence in noncriminal interactions where de-escalation and other interventions, other than criminalization and confinement, could be implemented.

Current data do not allow for a thorough and careful examination of these mechanisms, yet the geographical findings highlight how structural facets of governance and jurisdictional overlap *may* influence exposure to fatal police violence by law enforcement. Therefore, the application of these findings demands careful attention to the broader social and cultural significance of the unique understanding of reservation lands.

The PNAS paper “Heightened risk of fatal police violence in and around reservations for American Indian and Alaska Native Peoples in the United States” by Schwartz et al. (4) begins to fill this important gap and increases visibility of police violence for AIAN peoples living on or near reservations.

## Avoiding Misinterpretation.

One potential misinterpretation of these findings would be to suggest that reservations themselves are inherently risky or harmful environments. This would be an oversimplification that overlooks the profound cultural, social, and political importance of AIAN homelands, which may also be perceived as sources of strength and protection. At the governance level, tribal sovereignty has been a critical factor in supporting local community needs and in enabling rapid responses, thereby supporting public health outcomes (12). On an individual level, there are also many benefits to being on tribal homelands. Rich cultural and traditional practices, including kinship networks, make interpersonal relationships particularly vital in AIAN communities. In reservation communities, geographic isolation and lack of resources make social relations (i.e., peer, family, kinship, and community) a salient fixture of AIAN culture and survival. Historically, the physical, spiritual, and economic well-being of AIAN communities was intertwined with family and community relationships. Historically, many AIAN societies were collections of families whose relationships defined accepted duties and obligations to one another to ensure that communities thrived (13). These worldviews persist today (14). Coupled with the ability to live off the land, engage in cultural practices, and language, cements the importance of reservation life for many AIAN peoples.

Rather than interpreting these findings to suggest that AIAN individuals would be better off leaving these traditional homelands, these findings should prompt the reader to the structural factors that shape safety and well-being. If anything, the authors’ findings highlight the need to improve resource allocations, data systems, and policies that support tribal communities. If adequately addressed, it is reasonable to assume that public health and safety outcomes, including law enforcement fatal violence, would improve. Therefore, I caution readers to view the findings presented by Schwartz et al. not only as a contribution of new empirical evidence regarding the role of geography and police violence but also as a foundation for future research and policy efforts.

## Implications for Research and Policy.

I echo previous calls for urgent improvements to public health surveillance systems that are nationally representative and account for and respect tribal sovereignty. Improved surveillance systems would enable scientific inquiry and public health response to understand the full scope of police violence affecting AIAN communities and surrounding areas. Furthermore, improved surveillance systems would provide valuable insights for potential interventions.

More work is needed to improve the accuracy of AIAN identifications in national databases. This work also provides justification for the disproportionate harms affecting AIAN communities, warranting oversampling of AIAN communities in national databases to enable rigorous scientific inquiry. Relatedly, improved data would provide opportunities for future research to build on the geographic insights provided here by examining how jurisdictional arrangements, local policies, and social determinants shape patterns of police encounters in Indigenous communities. Advances in spatial analysis and multilevel modeling may provide new tools for understanding how overlapping governance systems influence risk. Improved data would also foster interdisciplinary approaches that can successfully link policing, public health, and governance studies–a complex, but necessary next step, as these do not exist in silos.

Increased data and visibility should translate into policy attention and increased resources for tribal communities. As this commentary cautions readers to see the strength and resilience in tribal communities, investments in community-based public safety initiatives that move away from reliance on law enforcement may help reduce situations in which law enforcement becomes the default responder to complicated public health challenges, allowing them to focus on the public safety concerns they are best equipped and trained to address.

## Conclusions

Documenting the heightened risk of fatal police violence for AIAN individuals, particularly AIAN men, on or near reservations is a long-overdue contribution to the field. Schwartz et al. shed light on a crucial yet often overlooked and understudied aspect of public health in the United States. Their research underscores the importance of rigorous methodological approaches in highlighting disparities affecting an often-invisible population. It serves as a reminder that police violence is influenced not only by individual characteristics but also by geographical, governance, and jurisdictional factors. Moreover, it highlights the potential of tribal communities to address these issues if the necessary resources are allocated to support tribal sovereignty and community-based initiatives. Reservations do not inherently, or solely, pose a risk or danger; they often serve as a source of strength and resilience when adequately supported.
